# Facial soft tissue thicknesses of Azerbaijan adult population: CT study

**DOI:** 10.1371/journal.pone.0348124

**Published:** 2026-06-05

**Authors:** Nigar Sultanova, Rasim Bayramov, Narmin Maharammova

**Affiliations:** 1 Department of Oral and Maxillofacial Surgery, Azerbaijan Medical University, Baku, Azerbaijan; 2 Department of Radiology, Azerbaijan Medical University, Baku, Azerbaijan; University of Perugia: Universita degli Studi di Perugia, ITALY

## Abstract

The aim of this study was to create a facial soft tissue thicknesses database of Azerbaijan adult population and find relation between FSTT and sex, age, body mass index (BMI). The study investigates the FSTT on 300 CT scans of living inviduals of three age groups: I, 18–25 years old; II, 26–45 years old; and III, 46 years and older. The CT images of the patients were divided into two groups according to BMI. The soft tissue thicknesses were measured at 20 landmarks, 10 along the midline and 10 bilateral. The mean, standard deviations, range, median for each anthropometric landmark were determined, and differences related to age, sex, BMI were calculated. This article presents the first database of facial soft tissues thicknesses of Transcaucasia populations.

## Introduction

Facial soft tissue thickness (FSTT) data are an important component of the facial approximation process and play a pivotal role in the planning of reconstructive and aesthetic surgeries of the craniofacial complex [1–3].

Over the past 140 years, more than 100 FSTT studies have been conducted on adults [4–6], following the same basic principles as established in 1883 by Welcker [7,8]. During the last years, a considerable volume of new data has been added [8], which is explained by the need to study the parameters of the modern population [9,10]. Studies conducted in scientific centers have confirmed that age, sex, ethnicity and body mass index (BMI) are key factors determining FSTT [11–18].

According to the data of Scopus, PubMed, and Google Scholar, study of the FSTT of different populations is described in the scientific literature. The populations showed notable differences in facial tissue thickness, raising the question of whether soft tissue thickness data from one population can be used for facial reconstruction in people of different origins [19–22, 23, 24].

A FSTT database was created for Australian [19], Caucasian [25–27], English [28], Bulgarian [29], Italian [30], German [31], Portuguese [32], Russian [33], Slovak [34, 35, 36], Czech [37], French [38], Egyptian [20], Turk [39–40], Iranian [41], Pakistani [42], Saudi [43], Brazilian [44–46], Colombian [15], Sri Lankan [47], Indian [23,48,49], Chinese [50], Korean [51], Japanese [24,52], Sudanese [53], Zulus [54], South African [55] and Black American [4] populations.

A literature search of the FSTT database of various populations showed no studies investigating the parameters of the Azerbaijan population.

This study aimed to create a facial soft tissue thicknesses database of Azerbaijan adult population and find relation between FSTT and sex, age, body mass index.

## Materials and methods

The study was approved by the Ethics Committee of Azerbaijan Medical University in accordance with the Code of Ethics of the World Medical Association and the Declaration of Helsinki. None of the patients was exposed to ionizing radiation for the purpose of this research.

The study of the facial soft tissues thickness of the Azerbaijan population was conducted using computed tomography (CT) images of the head of 300 patients (157 males and 143 females) aged 18–73 years who underwent diagnostic examination at the Department of Radiology of the Azerbaijan Medical University. The exclusion criteria were adentia, injuries, deformities of the craniofacial complex, history of reconstructive and aesthetic surgeries on the face and injection of fillers. The 200 CT-scans of the sample cover the entire face, the 100 CT-scans only the superior and middle part of the patient’s face. Scientific research was conducted between 2021 and 2023. The sample is representative of Azerbaijan population.

Males and females were classified according to their ages and BMI. The 300 subjects were divided into three age groups: I, 18–25 years old; II, 26–45 years old; and III, 46 years and older. During the examination, the 200 patients’ height and weight were measured, and their BMI was calculated (weight to height ratio [kg/m2]). The CT images of the patients were divided into two groups according to the following scale: BMI < 25 (underweight: BMI < 18.5; normal weight: BMI 18.6–24.9) and BMI > 25 (overweight: BMI 25.0–29.9; obesity: BMI ≥ 30.0).

### Measurements

The distance between the bone and soft tissue was measured using 20 classical anthropometric landmarks, 10 along the midline and 10 bilaterally, by Rhine and Campbell [4]. The craniometric landmarks, their synonyms and the description of the measurement direction are presented in Table 1.

**Table 1:** The craniometric landmarks, their synonyms and the description of the measurement direction.

Landmark		Description	Synonyms
**Midline landmarks**			
*Supraglabella*	sg-sg’	*Most anterior midline landmark on the frontal bone to the corresponding soft tissue*	**–**
*Glabella*	g-g’	*Crosslandmark between midline and supraorbital line to the corresponding soft tissue*	**–**
*Nasion*	n-n’	*Midlandmark on the nasofrontal suture to the corresponding soft tissue*	**–**
*Rhinion*	rhi-rhi’	*Midlandmark at the inferior free end on the internasal suture to the corresponding soft tissue*	**–**
*Mid-philtrum*	mp-mp’	*Midlandmark between the base of the nasal spine and prosthion on the anterior edge of the maxillae to the corresponding soft tissue*	subspinale
*Supradentale*	sd-sd’	*Most anterior landmark of the alveolar process of the maxilla to the corresponding soft tissue*	prosthion, labrale superius
*Infradentale*	id-id’	*Most anterior landmark of the alveolar process of the mandible to the corresponding soft tissue*	labrale inferius
*Mentolabial sulcus*	mls-mls’	*Deepest midlandmark in the groove superior to the mental eminence to the corresponding soft tissue*	labiomentale, supramentale
*Pogonion*	pg-pg’	*Most prominent midline landmark on the mental eminence of the mandible to the corresponding soft tissue*	mental eminence
*Gnathion*	gn-gn’	*Most inferior midline landmark at the mental symphysis of the mandible to the corresponding soft tissue*	menton, beneath chin
**Bilateral landmarks**			
*Frontal eminence*	fe-fe’	*Most anterior landmark of the forehead, centered on eyepupil, to the corresponding soft tissue*	–
*Supraorbital*	so-so’	*Most superior landmark at the supraorbital margin to the corresponding soft tissue*	supraconchion
*Infraorbital*	io-io’	*Most inferior landmark at the infraorbital margin to the corresponding soft tissue*	orbitale
*Inferior malar*	im-im’	*The deepest landmark at fossa canina to the corresponding soft tissue*	canina fossa
*Zygion*	zy-zy’	*Most lateral landmark of the zygomatic arch to the corresponding soft tissue*	zygomatic arch
*Condylion*	cdl-cdl’	*Most lateral landmark of the condylar process of the mandible to the corresponding soft tissue*	supraglenoid
*Gonion*	go-go’	*Landmark on the lateral border of mandible angle to the corresponding soft tissue*	–
*Supra M2*	sM2-sM2’	*Landmark on superior alveolar ridge above to the crown of the maxillary second molar to the corresponding soft tissue*	ectomolare
*Occlusal line*	ocl-ocl’	*Landmark at the mandibular ramus in the plane of dental occlusion to the corresponding soft tissue*	mid-ramus, mid-masseter
*Infra M2*	iM2-iM2’	*Landmark on inferior alveolar ridge bellow to the crown of the mandible second molar to the corresponding soft tissue*	inframolare, Sub M2

Midline anthropometric landmarks: 1. supraglabella (sg); 2. glabella (g); 3. nasion (n); 4. rhinion (rhi); 5. mid-philtrum (mp); 6. supradentale (sd); 7. infradentale (id); 8. mentolabial sulcus (mls); 9. pogonion (pg); 10. gnathion (gn).

Bilateral anthropometric landmarks: 1. frontal eminence (fe); 2. supraorbital (so); 3. infraorbital (io); 4. inferior malar (im); 5. zygion (zy); 6. condylion (cdl); 7. gonion (go); 8. supraM2 (sM2); 9. occlusal line (ocl); 10. infraM2 (iM2).

Head scan was performed using a Toshiba Aquilion CXL 128 CT scanner. The imaging protocol of 200 patients had a field of view of 24 cm x 19 cm, which enabled visualisation of all the included anatomical structures and landmarks from the supraglabella to the gnathion along the vertical axis and bilaterally to the condylion. At the same time, the imaging protocol of the other 100 patients allowed the inclusion of all landmarks except: infradentale, mentolabial sulcus, pogonion, gnation, frontal eminence, gonion, infraM2. Axial, sagittal and coronal CT views were collected for all subjects. The obtained images were converted into Digital Imaging and Communication in Medicine files and transferred to a personal portable computer (MDview 232; NEC, Germany). The image’s slice was 0.5 mm thickness. The skull images were positioned with the Frankfurt Horizontal Plane, and measurements were performed on the monitor by a cursor with an accuracy of 0.01 mm.

**Fig 1:**
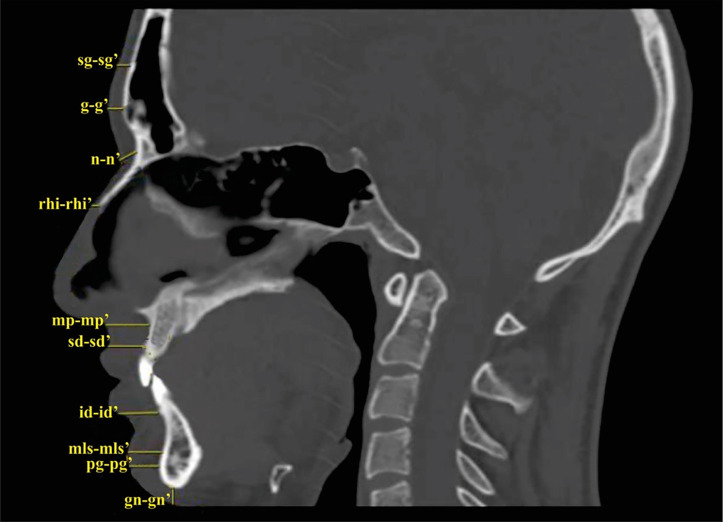
Anthropometric landmarks: 1-supraglabella (sg-sg’), 2-glabella (g-g’), 3-nasion (n-n’), 4-rhinion (rhi-rhi’), 5-mid-philtrum (mp-mp’), 6-supradentale (sd-sd’), 7- infradentale (id-id’), 8-mentolabial sulcus (mls-mls’), 9-pogonion (pg-pg’), 10-gnation (gn-gn’).

The following head CT slices were selected to measure the soft tissue thickness at 20 landmarks:

Slice 1 – On the midsagittal slice (Fig.1), FSTT was determined in the area of the following landmarks: supraglabella (sg); glabella (g); nasion (n); rhinion (rhi); mid-philtrum (mp); supradentale (sd); infradentale (id); mentolabial sulcus (mls); pogonion (pg); gnathion (gn).

**Fig 2:**
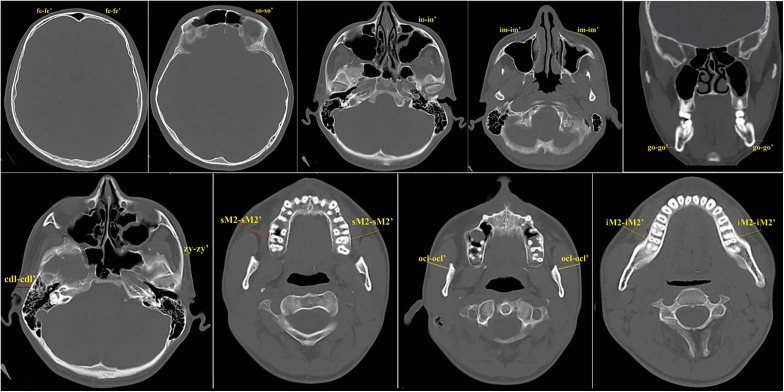
Anthropometric landmarks: 1-frontal eminence (fe-fe’), 2 -supraorbital (so-so’), 3 -infraorbital (io-io’), 4 -inferior malar (im-im’), 5 -gonion (go-go’), 6 -zygion (zy-zy’), 7 -condylion (cdl-cdl’), 8 -supra M2 (sM2-sM2’), 9 -occlusial line (ocl-ocl’), 10-infra M2 (iM2-iM2’).

Slices 2 – On the coronal slice (Fig. 2), FSTT was determined in the area of gonion (go).

Slices 3 – At different levels, 10 axial slices (Fig. 2), FSTT was determined in the area of: frontal eminence (fe); supraorbital (so); infraorbital (io); inferior malar (im); zygion (zy); condylion (cdl); supraM2 (sM2); occlusal line (ocl); infraM2 (iM2).

### Statistical analysis

The data were analyzed using the SPSS software version 26.0. (SPSS Inc., Chicago, IL). The intra-observer reliability agreement was achieved by repeat measurements the 30 landmarks of 30 randomly selected subjects with testing the intraclass correlation coefficient (ICC) for each point. The measurements were repeated in an interval of one week by the second author.

Since when testing the normality of the distribution of variation series using the Shapiro-Wilks test, in some series the 0-hypothesis was rejected, therefore nonparametric tests were used. Mann-Whitney U-test was applied to investigate the differences of soft tissue thickness between the sexes and BMI. Descriptive statistics of FSTT, classified by age and sex, age and BMI, sex and BMI was performed also by non-parametric Mann -Whithey U-test. The differences of FSTT between the three age groups were tested by using the Kruskal-Wallis H-test. Basic descriptive statistics, such as mean, standard deviation, range (between minimum and maximum), median and interquartile range (IQ) were calculated for each anthropometric landmark, taking into account BMI, sex and age. For all statistical analysis, differences were considered significant at *P* < 0.05.

## Results

The study included 300 participants, of whom 52.3% were males (N = 157) and 47.7% were females (N = 143). Their average age was 59 years with a standard deviation of 16.76. The youngest was 18 years old (females), and the oldest was 73 years old (males). The reliability and reproducibility of the method was determined using intra-class correlation coefficient (ICC). ICC showed significant consistency between the main and repeated records (Table 2). All raw data of FSTT required to replicate the results of this study are presented in S1Table.

**Table 2:** Method Error According to ICC (Intra-class Correlation Coefficient).

Landmark	ICC	95% CI	Landmark	ICC	95% CI
Supraglabella	0.998	0.995–0.999	Infraorbital L	1.000	0.999–1.000
Glabella	0.997	0.994–0.999	Inferiormalar R	1.000	0.999–1.000
Nasion	0.998	0.995–0.999	Inferiormalar L	1.000	0.999–1.000
Rhinion	0.987	0.974–0.994	Zygion R	1.000	0.999–1.000
Midphiltrum	1.000	0.999–1.000	Zygion L	1.000	1.000–1.000
Supradentale	0.999	0.999–1.000	Condylion R	1.000	0.999–1.000
Infradentale	1.000	0.999–1.000	Condylion L	1.000	0.999–1.000
Mentolabialsulcus	0.999	0.999–1.000	Gonion R	0.996	0.992–0.998
Pogonion	0.999	0.998–0.999	Gonion L	1.000	1.000–1.000
Gnation	1.000	0.999–1.000	SupraM2 R	1.000	1.000–1.000
Frontal eminence R	0.999	0.997–0.999	SupraM2 L	1.000	1.000–1.000
Frontal eminence L	0.998	0.997–0.999	Occlusal line R	1.000	0.999–1.000
Supraorbital R	0.998	0.997–0.999	Occlusal line L	1.000	0.999–1.000
Supraorbital L	0.998	0.994–0.999	Infra M2 R	1.000	1.000–1.000
Infraorbital R	0.999	0.999–1.000	Infra M2 L	1.000	0.999–1.000

Descriptive statistics of FSTT, classified by sex, are presented in Table 3. When the FSTT of males and females in the Azerbaijan population were compared, sexual dysmorphism was statistically confirmed (*P* < 0.05). The average values of FSTT in males exceeded those in females for all landmarks, with the exception of infraorbitale (r, l). Statistically significant differences were observed in 16 of the 20 landmarks, except for sg, g, sor (r), and zy (r,l). Differences of more than 2 mm between the average values of both sexes were observed in the mid-philtrum (+3.15 mm), supradentale (+2.65 mm) and gnathion (+2.21 mm).

**Table 3:** Descriptive statistics of the facial soft tissue thicknesses of Azerbaijan adult population, classified by sex in age-mixed group (all the measurements in millimeter).

Landmarks	Males						Females						
	N	Mean	SD	Min-Max	Median	IQ range	N	Mean	SD	Min-Max	Median	IQ range	P- Value
Supraglabella	157	5.78	1.11	3.2-9.3	5.7	4.9-6.6	143	5.68	1.24	3-9.1	5.5	4.8-6.4	0.298
Glabella	157	6.35	1.10	4.1-9.4	6.3	5.6-7.1	143	6.19	1.31	3.9-9.9	6.1	5.2-7	0.149
Nasion	157	8.03	1.47	4.1-12.3	7.8	7.1-9	143	6.66	1.35	3.7-10.4	6.6	5.7-7.5	0.000*
Rhinion	157	3.35	0.85	1.8-6	3.2	2.7-3.7	143	2.79	0.73	1.5-5.9	2.6	2.3-3.1	0.000*
Mid-philtrum	157	16.06	2.78	5.3-23.3	15.9	14.5-17.8	143	12.91	2.17	5.2-17.3	12.9	11.5-14.5	0.000*
Supradentale	157	12.52	1.99	8.9-19.3	12.3	11-13.8	143	9.87	1.74	6-16.6	9.6	8.7-10.8	0.000*
Infradentale	107	12.86	2.55	7.6-19.7	12.3	11.1-14.6	93	11.38	2.59	7.3-17.9	10.9	9.4-13.1	0.000*
Mentolabial sulcus	107	13.89	2.36	8.6-20	13.7	12.3-11.1	93	12.13	1.94	7.1-18.4	12	11-13.1	0.000*
Pogonion	107	12.29	2.21	7.8-17.4	12.4	10.8-13.7	93	10.95	2.19	6.8-20.1	10.9	9.5-12.3	0.000*
Gnation	107	9.20	2.57	3.8-18.5	9.2	7.4-10.7	93	6.99	2.24	3.2-14.6	6.5	5.5-7.9	0.000*
Frontal eminence (R)	107	4.84	1.66	2.6-15.5	4.5	3.8-5.6	93	4.08	1.38	2.3-10.5	3.8	3.3-4.5	0.000*
Frontal eminence (L)	107	4.75	1.63	2.6-14.8	4.3	3.6-5.4	93	3.98	1.43	2.3-10.9	3.7	3.2-4.3	0.000*
Supraorbital (R)	157	6.97	1.32	4-10.2	6.8	6-7.9	143	6.76	1.49	3.2-11.7	6.7	5.6-7.7	0.141
Supraorbital (L)	157	6.88	1.32	4-10.4	6.7	6-7.6	143	6.61	1.49	3.1-12.1	6.5	5.6-7.5	0.045*
Infraorbital (R)	157	5.33	1.64	2.9-10.4	5	4-6.1	143	5.92	2.06	2-13	5.6	4.6-6.7	0.010*
Infraorbital (L)	157	5.29	1.65	2.6-10.7	5	4-6.3	143	5.92	2.29	2-17.9	5.5	4.5-6.7	0.012*
Inferior malar (R)	157	14.53	3.24	5.3-23.3	14,8	12-16.8	143	13.63	2.85	7.4-19.2	13.7	11.4-16	0.012*
Inferior malar (L)	157	14.42	3.15	5.3-23.4	14,6	12.1-16.6	143	13.59	3.08	7.2-21.9	13.6	11.2-15.8	0.017*
Zygion (R)	157	12.28	3.32	4.7-21.2	12.5	9.5-14.6	143	11.62	2.84	5.6-20.2	11.1	9.8-13.6	0.060
Zygion (L)	157	12.48	3.42	4.9-21.9	12.5	9.8-14.6	143	11.81	3.01	6.3-20.9	11.5	9.6-14	0.072
Condylion (R)	157	13.64	2.47	7.6-20.7	13.5	12.2-15.2	143	12.58	2.36	7.3-19.2	12.4	10.9-14	0.000*
Condylion (L)	157	13.59	2.40	7.4-19.7	13.6	11.8-15.4	143	12.62	2,39	6.7-19	12.4	11-14.3	0.000*
Gonion (R)	107	15.88	4.78	4.5-28.5	15.9	12.3-19.3	93	14.43	3.81	7.5-24.4	14	11.6-17.2	0.017*
Gonion (L)	107	15.91	5.00	4.7-30.6	15.9	11.9-20.2	93	14.18	3.81	7.9-23.7	13.4	11.6-16.7	0.009*
SupraM2 (R)	157	29.42	5.07	16.9-41.4	29.9	25.9-33.1	143	28.34	4.88	15.8-40.7	28.1	25.5-31.4	0.030*
SupraM2 (L)	157	29.65	4.90	18.5-41.1	29.9	26.2-33.1	143	28.44	5.04	14.9-41.4	28.3	25.1-31.6	0.019*
Occlusal line (R)	157	23.82	3.59	15.6-34.2	23.9	21.3-26.3	143	22.94	3.06	16.1-32.5	22.9	21-24.6	0.040*
Occlusal line (L)	157	23.94	3.48	15.3-34.7	23.7	21.8-26.4	143	22.94	3.00	15-30.1	22.9	21.1-24.9	0.022*
InfraM2 (R)	107	23.63	4.50	12.5-32	23.9	21-26.8	93	21.88	3.79	12.2-31	22.1	19.9-24.1	0.002*
InfraM2 (L)	107	23.98	4.59	12.4-33.7	23.5	21.1-27.6	93	21.98	4.05	11.4-34.8	22	19.2-23.9	0.001*

SD -Standard Deviation; IQ-interquartile range; Mann-Whitnet U test; ^*^Significant (P < 0.05).

Descriptive statistics of FSTT, classified by age, are presented in Table 4. A comparison between the three age groups showed a statistically significant difference in FSTT in 14 of the 20 landmarks. Thickening of the facial soft tissues with age was observed in all the studied landmarks, except of the mid-philtrum and supradentale. In the area of these anthropometric landmarks, a gradual thinning of FSTT was observed. When age groups 1 and 2 were compared, an increase in soft tissue thickness of more than 2 mm was observed in the area of the zygion,l (+ 2.13 мм), gonion,r (+2.12мм), gonion,l (+2.52 мм). When age groups 1 and 3 were compared, an increase in soft tissue thickness by more than 2 mm was noted in the area of mid-philtrum (+ 2.27мм), zygion,r (+ 2.71мм), zygion,l (+ 2.99мм), condilion,r (+2.28 мм), condilion,l (+2.39 мм), gonion,r (+3.63 мм), gonion,l (+3.71мм), supra M2,r (+2.56 мм), supraM2,l (+2.4 мм).

**Table 4:** Descriptive statistics of the facial soft tissue thicknesses of Azerbaijan adult population, classified by age in sex-mixed group (all the measurements in millimeter).

Landmarks	18-25 years						26-45 years						≥ 46 years						P-value
	N	Mean	SD	Min-Max	Median	IQ range	N	Mean	SD	Min-Max	Median	IQ range	N	Mean	SD	Min-Max	Median	IQ range	
Supraglabella	49	5.34	0.94	3.5-8.1	5.1	4.7-5.8	147	5.57	1.09	3-9.3	5.4	4.8-6.2	104	6.15	1.26	3.2-9.1	6.1	5.3-7	0.000*
Glabella	49	5.99	1.10	4-8.4	6.1	5,5-6.7	147	6.12	1.14	3.9-9.1	6.1	5.1-7	104	6.62	1.27	3.9-9.9	6.55	5.75-7.45	0.002*
Nasion	49	7.33	1.57	4.2-11.6	7.1	6.3-8.1	147	7.25	1.64	3.7-12.3	7.1	6.1-8.4	104	7.58	1.46	4.3-11.1	7.5	6.6-8.8	0.188
Rhinion	49	2.79	0.64	1.5-4.3	2.7	2.3-3.2	147	2.98	0.83	1.8-5.9	2.8	2.4-3.5	104	3.37	0.87	1.8-6	3.2	2.7-3.75	0.000*
Mid-philtrum	49	15.89	2.67	9-23.3	15.7	14.2-17.1	147	14.77	2.99	5.2-23.2	14.7	12.8-16.9	104	13.62	2.76	8.2-21.1	13.3	11.75-15.45	0.000*
Supradentale	49	12.01	2.01	7.9-17.7	11.7	10.3-13.4	147	11.16	2.11	7.1-17	10.8	9.5-12.5	104	11.03	2.60	6-19.3	10.65	9.2-12.35	0.014*
Infradentale	33	11.85	2.57	7.8-18.1	11.9	9.5-13.9	97	12.19	2.71	7.3-19.7	11.6	10.4-13.8	70	12.31	2.68	7.6-18.6	12.05	10.3-14.3	0.706
Mentolabial sulcus	33	12.64	2.49	7.1-19.8	12.3	11.1-14	97	12.89	2.35	7.5-19.4	12.5	11.5-14.2	70	13.52	2.21	8.2-20	13.15	12.3-15.1	0.056
Pogonion	33	11.06	2.15	7-17.1	11.1	9.5-12.1	97	11.54	2.40	7.2-20.1	11.2	10-12.9	70	12.12	2.15	6.8-17.4	12.3	11-13	0.035*
Gnation	33	7.65	2.50	3.9-14.6	7.7	5.7-9.1	97	7.93	2.66	3.2-15.1	7.3	5.9-9.8	70	8.75	2.67	3.8-18.5	8.2	6.7-10.2	0.052
Frontal eminence (R)	33	4.08	0.94	2.3-7.3	3.9	3.6-4.6	97	4.47	1.70	2.3-15.5	4.1	3.6-4.9	70	4.71	1.61	2.4-10.5	4.4	3.7-5.5	0.185
Frontal eminence (L)	33	4.05	0.99	2.3-7.1	3.7	3.6-4.6	97	4.33	1.65	2.4-14.8	4	3.4-4.8	70	4.63	1.69	2.3-10.9	4.25	3.5-5.4	0.268
Supraorbital (R)	49	6.37	1.30	4.4-10.2	6.3	5.5-6.9	147	6.65	1.23	3.2-10.2	6.6	5.8-7.4	104	7.40	1.52	4.1-11.7	7.3	6.3-8.5	0.000*
Supraorbital (L)	49	6.22	1.38	4.1-10.1	6.1	5.3-6.8	147	6.56	1.21	3.1-10.2	6.5	5.7-7.2	104	7.29	1.52	4.2-12.1	7.2	6.1-8.3	0.000*
Infraorbital (R)	49	4.73	1.30	3.2-9	4.4	3.7-5.6	147	5.38	1.66	2.2-12.1	5.2	4.1-6.2	104	6.36	2.11	2-13	6	4.9-8	0.000*
Infraorbital (L)	49	4.70	1.35	2.6-8.8	4.4	3.6-5.4	147	5.33	1.64	2-11.7	5.1	4.2-6.2	104	6.38	2.42	2-17.9	6	4.7-7.85	0.000*
Inferior malar (R)	49	1.72	3.17	5.3-23.3	12.3	10.1-14.6	147	14.17	2.97	7.4-21.3	14.4	11.9-16.2	104	14.66	3.04	7.4-20	14.7	12.35-17.1	0.001*
Inferior malar (L)	49	12.80	3.21	5.3-23.4	12.7	10.4-14.7	147	14.02	2.97	7.2-21.2	14.2	11.7-16	104	14.60	3.20	7.2-21.9	14.35	12.05-16.9	0.005*
Zygion (R)	49	10.07	2.94	4.7-18.9	9.4	8.3-11.1	147	12.02	3.02	5.1-21.2	12	9.8-14	104	12.78	2.96	6.6-20.2	12.5	10.85-14.9	0.000*
Zygion (L)	49	10.08	2.96	5-19.1	9.5	8.1-10.9	147	12.21	3.11	4.9-21.9	12.2	9.6-14.2	104	13.07	3.13	6.3-20.9	12.5	11-15.15	0.000*
Condylion (R)	49	11.78	2.40	7.3-20.5	11.6	10.2-12.7	147	12.93	2.29	7.4-20.7	13	11.2-14.6	104	14.06	2.41	8.6-19.2	13.75	12.4-16.1	0.000*
Condylion (L)	49	11.71	2.25	7.6-19.7	11.3	10.2-12.8	147	12.91	2.25	6.7-17.8	12.9	11.3-14.8	104	14.10	2.41	9.5-19	13.6	12.4-16	0.000*
Gonion (R)	33	12.91	3.39	7.5-20.7	12.8	10.4-14.8	97	15.03	4.49	4.5-28.5	14.8	11.9-18	70	16.54	4.26	8.2-24.9	16.25	14-19.6	0.000*
Gonion (L)	33	12.59	3.47	7-20.5	11.9	9.9-15.5	97	15.11	4.88	4.7-30.6	14.14	11.8-18	70	16.30	4.10	9-24.7	16.35	13.3-19.7	0.000*
SupraM2 (R)	49	27.32	4.59	15.8-36.7	27	24.7-30.1	147	28.74	4.70	16.9-41.4	28.8	25.6-32.5	104	29.88	5.42	16.9-40.7	30.4	26.3-33.65	0.007*
SupraM2 (L)	49	27.69	4.64	14,9-37.3	27.7	25.2-30.6	147	28.81	4.68	16.1-41.1	29.1	25.5-32.2	104	30.09	5.41	18.2-41.4	30.6	25.9-34.05	0.017*
Occlusal line (R)	49	22.27	3.14	16.1-31.9	22	20-24	147	23.31	3.37	15.6-34.2	23.2	21-25.5	104	24.07	3.34	16.3-32.5	23.65	21.95-26.6	0.005*
Occlusal line (L)	49	22.24	3.14	15-31.5	22	20.7-23.7	147	23.46	3.31	15.3-34.7	23.4	21.6-25.4	104	24.04	3.21	16.3-32.8	23.85	21.9-26.55	0.003*
InfraM2 (R)	33	22.96	4.15	12.2-32	22.8	21.9-25.3	97	23.13	4.40	12.5-31.6	23.5	20.5-26.3	70	22.31	4.13	12.7-31.9	22.05	19.4-24.7	0.255
InfraM2 (L)	33	22.86	4.33	11.4-32.2	23.3	21.3-25.3	97	23.25	4.61	12-33.7	23.4	20-26.4	70	22.86	4.32	15.3-34.8	22	20-25.4	0.538

SD -Standard Deviation; IQ-interquartile range; Kruskal-Wallis H-test; ^*^Significant (P < 0.05).

Descriptive statistics of FSTT, classified by age and sex, are presented in Tables 5–7. Reliable differences were found in the middle and lower region of the face in 8 out of 20 anthropometric landmarks (P < 0.05) between the FSTT of males and females aged 18–25 years (Table 5). The tissue thickness of males at these landmarks was greater than that of females. Despite the absence of statistically significant differences in the remaining measured landmarks, the average values in males were higher than those in females, except for the infraorbital (r, l).

**Table 5:** Descriptive statistics of the facial soft tissue thicknesses of Azerbaijan adult population, classified by sex amoung I age group (18-25years) (all the measurements in millimeter).

Landmarks	Males 18–25 years							Females 18–25 years						
	N	Mean	SD	Min-Max	Median	IQ range	N		Mean	SD	Min-Max	Median	IQ range	P-Value
Supraglabella	34	5.41	0.92	4.1-8.1	5.2	4.8-5.8	15		5.18	1.01	3.5-7.3	5.1	4.5-5.8	0.565
Glabella	34	6.15	1.01	4.1-8.1	6.1	5.6-6.8	15		5.61	1.24	4-8.4	5.5	4.3-6.6	0.094
Nasion	34	7.84	1.52	5.1-11.6	7.65	6.8-8.8	15		6.18	0.96	4.2-8.1	6.4	5.5-6.7	0.000*
Rhinion	34	2.93	0.66	2-4.3	2.85	2.3-3.4	15		2.49	0.50	1.5-3.4	2.4	2.1-2.9	0.041*
Mid-philtrum	34	16.78	2.46	13.2-23.3	16.3	14.9-17.7	15		13.88	2.02	9-16.9	13.9	12.6-15.5	0.000*
Supradentale	34	12.72	1.85	9.3-17.7	13.05	11.2-14.2	15		10.39	1.34	7.9-13.3	10.1	9.6-11.3	0.000*
Infradentale	23	12.53	2.46	8.8-18.1	12.7	10.4-14.1	10		10.28	2.18	7.8-15.1	9.6	9.2-11.1	0.020*
Mentolabial sulcus	23	13.22	2.58	8.6-19.8	13.4	11.2-14.6	10		11.31	1.75	7.1-13.3	11.4	10.8-12.1	0.036*
Pogonion	23	11.40	2.22	8.1-17.1	11.3	9.8-13.4	10		10.29	1.85	7-13.4	10.5	9.5-11.5	0.210
Gnation	23	8.07	2.17	4.5-12.6	8.5	5.7-9.4	10		6.70	3.04	3.9-14.6	6.05	5.1-6.9	0.057
Frontal eminence (R)	23	4.20	0.95	3-7.3	4	3.6-4.6	10		3.79	0.91	2.3-5	3.8	3.5-4.6	0.492
Frontal eminence (L)	23	4.22	1.01	2.7-7.1	4	3.6-4.7	10		3.67	0.86	2.3-5.1	3.65	3.5-4.2	0.306
Supraorbital (R)	34	6.65	1.36	4.5-10.2	6.45	6-7.1	15		5.75	0.92	4.4-7.2	5.6	5.1-6.6	0.031*
Supraorbital (L)	34	6.54	1.44	4.1-10.1	6.3	5.8-7.1	15		5.49	0.91	4.2-7.1	5.4	4.7-6.1	0.009*
Infraorbital (R)	34	4.71	1.47	3.2-9	4.15	3.7-5.7	15		4.79	0.83	3.4-6	5	4-5.5	0.307
Infraorbital (L)	34	4.64	1.47	2.6-8.8	4.2	3.6-5.4	15		4.83	1.05	3.3-7.4	4.5	4.2-5.4	0.302
Inferior malar (R)	34	13.08	3.44	5.3-23.3	13.05	10.7-15	15		11.91	2.35	9-16.2	12	9.9-13.6	0.220
Inferior malar (L)	34	13.16	3.43	5.3-23.4	13.55	10.7-15	15		11.99	2.58	8.9-17.2	11.4	9.4-14.6	0.197
Zygion (R)	34	10.11	3.30	4.7-18.8	9.2	8.1-11.2	15		9.98	2.00	6.2-13.8	10.5	9-11.1	0.508
Zygion (L)	34	10.17	3.23	5-19.1	9.4	8.3-10.8	15		9.88	2.30	6.3-14.8	10.4	7.9-11.3	0.922
Condylion (R)	34	12.27	2.40	8-20.5	12.05	10.6-12.8	15		10.65	2.04	7.3-15	9.9	9.5-12	0.013*
Condylion (L)	34	12.21	2.26	7.9-19.7	11.75	10.9-12.9	15		10.59	1.84	7.6-14.4	10	9.6-11.9	0.013*
Gonion (R)	23	13,23	3.49	7.8-20.7	12.8	10.4-14.8	10		12.17	3.19	7.5-17.2	12	9.2-15.3	0.531
Gonion (L)	23	12.87	3.58	7-20.5	12.4	10.2-16.7	10		11,94	3.31	7.9-16.4	11.8	8.4-15.5	0.400
SupraM2 (R)	34	28.09	4.45	20.1-36.7	27	24.9-30.8	15		25.59	4.58	15.8-32.8	26.6	23.3-27.7	0.216
SupraM2 (L)	34	28.37	4.26	20.6-37.3	27.85	25.2-30.8	15		26.16	5.25	14.9-32.4	26.6	23.8-30.6	0.318
Occlusal line (R)	34	22.58	3.26	16.9-31.9	22.05	20-24.3	15		21.56	2.85	16.1-28	21.5	19.9-22.8	0.345
Occlusal line (L)	34	22.70	3.07	17.3-31.5	22.2	20.7-24	15		21.21	3.13	15-27.2	21.8	19.9-23.4	0.175
InfraM2 (R)	23	23.37	4.06	15.7-32	22.6	21.9-25.7	10		22.04	4.43	12.2-28.4	22.85	20.4-24	0.769
InfraM2 (L)	23	23.17	4.32	15.5-32.2	23.1	20.2-26	10		22.15	4.51	11.4-27.8	23.4	21.6-24	0.860

SD -Standard Deviation; IQ-interquartile range; Mann-Whitnet U test; ^*^Significant (P < 0.05).

**Table 6:** Descriptive statistics of the facial soft tissue thicknesses of Azerbaijan adult population, classified by sex amoung II age group (26-45 years) (all the measurements in millimeter).

Landmarks	Males 26–45 years						Females 26–45 years						
	N	Mean	SD	Min-Max	Median	IQ range	N	Mean	SD	Min-Max	Median	IQ range	P-value
Supraglabella	80	5.74	1.07	4-9.3	5.7	4.85-6.5	67	5.37	1.09	3-8.9	5.2	4.7-5.9	0.028^*^
Glabella	80	6.35	1.05	4.2-8.6	6.25	5.5-7.3	67	5.86	1.21	3.9-9.1	5.6	5-6.7	0.006*
Nasion	80	8.09	1.46	4.1-12.3	8.05	7.1-9.1	67	6.25	1.23	3.7-10	6.2	5.4-6.8	0.000*
Rhinion	80	3.39	0.85	2.1-5.9	3.35	2.7-3.75	67	2.50	0.46	1.8-3.8	2.4	2.1-2.8	0.000*
Mid-philtrum	80	16.21	2.81	5.3-23.2	16	14.6-18.25	67	13.06	2.20	5.2-17.3	13.3	11.5-14.7	0.000*
Supradentale	80	12.39	1.84	8.9-17	12.2	11-13.55	67	9.69	1.33	7.1-13.2	9.5	8.7-10.6	0.000*
Infradentale	52	12.98	2.57	7.6-19.7	12.25	11.35-14.8	45	11.28	2.60	7.3-17.9	10.4	9.6-12.7	0.000*
Mentolabial sulcus	52	13.93	2.33	8.6-19.4	13.65	12.25-15.55	45	11.70	1.76	7.5-18.4	11.5	10.8-12.3	0.000*
Pogonion	52	12.53	2.17	7.8-16.5	12.55	10.95-14.1	45	10.40	2.16	7.2-20.1	10.1	9.1-11.2	0.000*
Gnation	52	9.30	2.52	3.8-15.1	9.25	7.45-11.1	45	6.35	1.82	3.2-11.5	6.1	5.2-7.2	0.000*
Frontal eminence (R)	52	5.12	1.98	2.8-15.5	4.7	4-5.7	45	3.72	0.84	2.3-6.7	3.6	3.2-4	0.000*
Frontal eminence (L)	52	4.98	1.92	2.8-14.8	4.5	3.95-5.4	45	3.59	0.81	2.4-6.6	3.6	3.1-3.9	0.000*
Supraorbital (R)	80	7.00	1.25	4-10.2	6.9	6.1-7.9	67	6.23	1.08	3.2-8.7	6.2	5.4-6.9	0.000*
Supraorbital (L)	80	6.91	1.24	4-10.2	6.85	6.05-7.7	67	6.14	1.04	3.1-8.5	6.1	5.5-6.8	0.000*
Infraorbital (R)	80	5.39	1.61	2.9-10.4	5.2	4.1-6.2	67	5.36	1.74	2.2-12.1	5.2	4.2-6.2	0.980
Infraorbital (L)	80	5.34	1.56	2.9-10.7	5.1	4.15-6.3	67	5.32	1.74	2-11.7	5.2	4.3-6	0.994
Inferior malar (R)	80	14.92	2.96	7.4-21.3	15.55	12.45-16.65	67	13.27	2.74	7.4-19.2	13.7	11.4-15.1	0.001*
Inferior malar (L)	80	14.74	2.90	8.4-21.2	15	12.7-16.85	67	13.15	2.84	7.2-20.8	13.7	11.4-15.2	0.001*
Zygion (R)	80	13.03	3.00	5.1-21.2	13.2	11.3-14.9	67	10.82	2.60	5.6-19.2	10.3	9.4-12.2	0.000*
Zygion (L)	80	13.27	3.13	4.9-21.9	13.45	11.2-14.75	67	10.96	2.61	6.6-19	10.5	9.1-12.8	0.000*
Condylion (R)	80	13.73	2.28	7.6-20.7	13.9	12.45-15.1	67	11.97	1.90	7.4-16.1	11.8	10.8-13.3	0.000*
Condylion (L)	80	13.67	2.18	7.4-17.8	13.95	12.6-15.3	67	12.01	1.99	6.7-16.2	12	10.9-13.4	0.000*
Gonion (R)	52	16.52	4.93	4.5-28.5	16.35	13.7-20.25	45	13.30	3.20	8.4-22.6	12.7	11.2-14.9	0.000*
Gonion (L)	52	16.77	5.35	4.7-30.6	17.15	13.25-20.75	45	13.19	3.43	8-22.5	12.3	10.7-14.7	0.000*
SupraM2 (R)	80	29.78	5.01	16.9-41.4	30.5	27.25-33.3	67	27.50	3.98	17-38.1	27.3	25-29.4	0.001*
SupraM2 (L)	80	29.92	4.67	18.5-41.1	30.55	27.3-32.8	67	27.49	4.37	16.1-40.4	27.1	24.6-29.4	0.000*
Occlusal line (R)	80	24.31	3.52	15.6-34.2	24.15	21.9-26.35	67	22.11	2.77	16.6-28	22.3	20.3-24	0.000*
Occlusal line (L)	80	24.53	3.46	15.3-34.7	24.25	22.05-27.2	67	22.20	2.63	16-28	22.4	19.9-24.2	0.000*
InfraM2 (R)	52	24.38	4.53	12.5-31.6	25.65	21.65-27.1	45	21.70	3.82	13.7-29.2	21.6	19.7-24.3	0.001*
InfraM2 (L)	52	24.75	4.61	12.4-33.7	25.45	22.1-28.15	45	21.52	4.00	12-29.9	22	18.6-23.9	0.000*

SD -Standard Deviation; IQ-interquartile range; Mann-Whitnet U test; ^*^Significant (P < 0.05)

**Table 7:** Descriptive statistics of the facial soft tissue thicknesses of Azerbaijan adult population, classified by sex amoung III age group (46 and above) (all the measurements in millimeter).

Landmarks	Males ≥ 46 years						Females ≥ 46 years						P-value
	N	Mean	SD	Min-Max	Median	IQ range	N	Mean	SD	Min-Max	Median	IQ range	
Supraglabella	43	6.15	1.21	3.2-9	6.1	5.3-6.9	61	6.15	1.30	3.6-9.1	6	5.1-7	0.810
Glabella	43	6.52	1.24	4.1-9.4	6.5	5.6-7.4	61	6.69	1.29	3.9-9.9	6.6	5.8-7.5	0.566
Nasion	43	8.06	1.46	4.3-11.1	8.1	6.9-9.1	61	7.23	1.37	4.4-10.4	7.1	6.4-8.3	0.004*
Rhinion	43	3.62	0.87	1.8-6	3.5	3-4.2	61	3.19	0.82	1.9-5.9	3	2.6-3.7	0.006*
Mid-philtrum	43	15.21	2.82	8.4-21.1	15.3	13.2-17	61	12.50	2.11	8.2-17.1	12.6	11.2-13.9	0.000*
Supradentale	43	12.59	2.37	9.2-19.3	12.2	10.6-14.9	61	9.93	2.18	6-16.6	9.4	8.2-11.2	0.000*
Infradentale	32	12.92	2.64	8.2-18.6	12.25	11.2-15	38	11.79	2.63	7.6-17.2	11.4	9.4-14.1	0.106
Mentolabial sulcus	32	14.31	2.23	10.3-20	14.55	12.7-15.8	38	12.86	1.98	8.2-17.3	12.85	11.7-14.3	0.007*
Pogonion	32	12.53	2.19	8.1-17.4	12.5	11.5-13.8	38	11.77	2.09	6.8-16.8	12.2	10.4-12.9	0.177
Gnation	32	9.84	2.73	5.6-18.5	9.65	7.8-10.9	38	7.83	2.26	3.8-14.3	7.3	6.4-9.3	0.001*
Frontal eminence (R)	32	4.84	1.37	2.6-7.8	4.85	3.8-6	38	4.59	1.80	2.4-10.5	4.35	3.3-5.3	0.229
Frontal eminence (L)	32	4.75	1.43	2.6-8.2	4.65	3.5-5.9	38	4.53	1.90	2.3-10.9	4.05	3.4-5.2	0.229
Supraorbital (R)	43	7.15	1.39	4.1-10.1	6.9	6.1-8.2	61	7.58	1.59	4.6-11.7	7.8	6.6-8.5	0.171
Supraorbital (L)	43	7.12	1.34	4.2-10.4	7.2	6.1-7.8	61	7.40	1.64	4.2-12.1	7.3	6.2-8.4	0.422
Infraorbital (R)	43	5.72	1.71	3-9.9	5.4	4.6-6.8	61	6.82	2.26	2-13	6.2	5.3-8.4	0.013*
Infraorbital (L)	43	5.71	1.81	3.1-9.7	5.7	4-6.8	61	6.85	2.69	2-17.9	6.2	5.3-8.2	0.029*
Inferior malar (R)	43	14.96	3.32	7.4-20	15.4	12.5-18.2	61	14.45	2.84	7.7-19.2	14.6	12.3-16.6	0.337
Inferior malar (L)	43	14.80	3.20	7.2-21.4	14.6	12.3-18	61	14.46	3.23	8.9-21.9	14.1	11.7-16.5	0.505
Zygion (R)	43	12.62	3.24	6.6-19.7	12.5	10.6-15.4	61	12.90	2.76	7.3-20.2	12.4	10.9-14.9	0.712
Zygion (L)	43	12.86	3.34	6.3-20.1	12.5	10.7-15	61	13.23	2.99	7.3-20.9	12.6	11.2-15.3	0.627
Condylion (R)	43	14.54	2.43	8.6-18.6	15	12.8-16.5	61	13.73	2.36	9-19.2	13.2	12.4-15.1	0.048*
Condylion (L)	43	14.54	2.44	9.5-18.7	14.4	12.5-16.3	61	13.79	2.36	9.6-19	13.4	12.3-15.2	0.106
Gonion (R)	32	16.76	4.78	8.2-24.9	16.45	12.7-20.8	38	16.36	3.82	8.5-24.4	16.05	14-18.7	0.684
Gonion (L)	32	16.72	4.55	9-24.7	17.45	13.1-20.5	38	15.94	3.70	9.1-23.7	15.5	13.3-19	0.358
SupraM2 (R)	43	29.79	5.55	17.9-40.2	30.4	25.4-34.5	61	29.94	5.36	16.9-40.7	29.9	26.9-32.6	0.939
SupraM2 (L)	43	30.17	5.67	20-41.1	32.1	24.9-34.4	61	30.04	5.26	18.2-41.4	30.4	26.1-33.5	0.828
Occlusal line (R)	43	23.89	3.81	16.3-32.2	24	20.9-26.8	61	24.19	2.99	17.1-32.5	23.6	22.2-26.3	0.663
Occlusal line (L)	43	23.85	3.64	16.3-32.8	23.4	21.8-26.6	61	24.18	2.90	17.1-30.1	24.1	21.9-26.5	0.588
InfraM2 (R)	32	22.62	4.65	12.7-31.9	22.4	18.8-26.4	38	22.06	3.67	15.6-31	22	20-24.1	0.592
InfraM2 (L)	32	23.30	4.67	15.4-33.7	22.75	19.8-26.3	38	22.49	4.03	15.3-34.8	21.8	20-23.8	0.426

SD -Standard Deviation; IQ-interquartile range; Mann-Whitnet U test; ^*^Significant (P < 0.05).

Reliable differences were found in the upper, middle, and lower parts of the face in 19 out of 20 landmarks (P < 0.05) when comparing the FSTT values of males and females aged 26–45 years (Table 6). The values in males were considerably higher at these landmarks. Notably, tissue thickness in the infraorbital (r, l) landmarks was almost the same in both sexes.

Significant differences were observed in the middle and lower part of the face in the area of 8 of the 20 landmarks in males and females aged 46 years and above (*P* < 0.05) (Table 7). The FSTT at these landmarks in males of this age group was greater than those in females. Concurrently, the tissue thickness in the infraorbital, glabella, supraorbital, zygion and occlusal line landmarks were higher in females than in males.

Descriptive statistics of FSTT, classified by BMI, are presented in Tables 8. Since the division into several subgroups by BMI reduces the number of elements in the subgroups, the confidence intervals expand and thus the obtained statistical results become unreliable, based on this, it was decided to distribute the subjects into two categories with a BMI greater than and less than 25. Statistically significant differences in the FSTT between the two groups were observed in 17 of the 20 anthropometric landmarks, whereas no difference was observed in the area of some mid-line parameters (mid-philtrum, supradentale, infradentale). In individuals with overweight and obesity, FSTT greater than 3 mm was determined in the following areas: zygion,r (+ 3.57мм), zygion,l (+ 3.83мм), gonion,r (+ 5.05мм), gonion,l (+ 5.49мм), supraM2,r (+ 4.13мм), supraM2,l (+ 4.36мм), occlusial line,r (+ 3.37мм), occlusial line,l (+ 3.50мм), infra M2,l (+ 3.03мм).

**Table 8:** Descriptive statistics of the facial soft tissue thicknesses of Azerbaijan adult population, classified by BMI in sex-mixed group (all the measurements in millimeter).

Landmarks	< 25 kg/m2						≥ 25 kg/m2						P-value
	N	Mean	SD	Min-Max	Median	IQ range	N	Mean	SD	Min-Max	Median	IQ range	
Supraglabella	91	5.17	0.98	3.2-8.9	5.1	4.5-5.5	109	6.00	1.15	3-9.3	5.9	5.1-6.9	0.000*
Glabella	91	5.68	1.13	3.9-9.1	5.4	4.9-6.5	109	6.39	1.10	4-8.9	6.3	5.6-7.1	0.000*
Nasion	91	6.70	1.47	3.7-11.2	6.5	5.6-7.7	109	7.72	1.57	4.9-12.3	7.5	6.6-8.9	0.000*
Rhinion	91	2.71	0.64	1.5-4.5	2.6	2.3-3	109	3.36	0.91	1.8-6	3.2	2.7-3.7	0.000*
Mid-philtrum	91	14.36	2.74	5.3-20.4	14.2	12.6-16.3	109	14.34	2.90	8.2-20.7	14.6	12.1-16	0.863
Supradentale	91	10.91	2.24	7-17	10.7	9.1-12.2	109	11.45	2.34	6-16.8	11	9.7-13.1	0.094
Infradentale	91	12.00	2.72	7.3-18.6	11.5	9.8-14	109	12.32	2.62	7.6-19.7	11.9	10.5-14.1	0.340
Mentolabial sulcus	91	12.16	2.05	7.1-17.4	11.9	10.9-13.3	109	13.83	2.30	9.1-20	13.4	12.3-15.4	0.000*
Pogonion	91	10.58	1.82	6.8-15.8	10.6	9.1-11.7	109	12.57	2.27	7.2-20.1	12.5	11.5-13.8	0.000*
Gnation	91	6.78	1.86	3.8-12.4	6.5	5.5-7.7	109	9.33	2.67	3.2-18.5	9.3	7.5-10.8	0.000*
Frontal eminence (R)	91	3.95	1.49	2.3-15.5	3.8	3.2-4.3	109	4.94	1.51	2.4-10.5	4.6	3.8-5.7	0.000*
Frontal eminence (L)	91	3.89	1.49	2.3-14.8	3.6	3.2-4.2	109	4.81	155	2.3-10.9	4.4	3.8-5.6	0.000*
Supraorbital (R)	91	6.15	1.09	3.2-10	6.1	5.3-6.8	109	7.55	1.39	4.6-11.7	7.5	6.6-8.5	0.000*
Supraorbital (L)	91	5.99	1.11	3.1-9.5	6	5.2-6.6	109	7.42	1.38	4.4-12.1	7.2	6.5-8.3	0.000*
Infraorbital (R)	91	4.80	1.47	2-10.4	4.5	3.8-5.5	109	6.24	1.90	3-13	5.8	4.9-7.1	0.000*
Infraorbital (L)	91	4.71	1.43	2-10.7	4.5	3.7-5.4	109	6.18	2.18	3.1-17.9	5.7	4.7-7.1	0.000*
Inferior malar (R)	91	12.38	2.61	5.3-18.7	12.3	10.3-14.4	109	14.89	3.02	7.7-20.5	15.1	12.8-17.3	0.000*
Inferior malar (L)	91	12.31	2.68	5.3-18.9	12.6	10.3-14.2	109	14.72	3.12	7.2-21.9	14.9	12.3-17.2	0.000*
Zygion (R)	91	9.66	2.28	4.7-15.9	9.4	8.2-11	109	13.23	2.66	7.9-21.2	13	11.3-14.9	0.000*
Zygion (L)	91	9.72	2.25	4.9-15.9	9.5	8.3-11	109	13.55	2.92	7.7-21.9	13.3	11.5-15	0.000*
Condylion (R)	91	11.45	1.68	7.6-15.7	11.6	10.2-12.5	109	14.32	2.12	9-20.7	14.3	12.8-16.1	0.000*
Condylion (L)	91	11.50	1.77	7.5-16.1	11.4	10.2-12.5	109	14.15	2.04	10.1-18.9	14.3	12.7-15.5	0.000*
Gonion (R)	91	12.46	3.32	4.5-22.1	12.4	10.3-14.3	109	17.50	3.86	8.9-28.5	17.6	15.1-20.4	0.000*
Gonion (L)	91	12.11	3.06	4.7-20.8	11.9	9.9-13.8	109	17.61	4.08	8.4-30.6	17.8	14.7-20.5	0.000*
SupraM2 (R)	91	25.75	4.33	15.8-41.4	25.6	23.5-27.8	109	29.89	4.60	17.3-40	30.4	27.3-33.1	0.000*
SupraM2 (L)	91	25.82	4.41	14.9-41.1	25.5	23.6-27.9	109	30.18	4.53	18.7-41.4	30.5	27.1-33.4	0.000*
Occlusal line (R)	91	21.35	2.82	15.6-28.8	21.6	19.3-23.3	109	24.72	3.31	18-34.2	24.6	22.6-26.8	0.000*
Occlusal line (L)	91	21.29	2.75	15-28.7	21.7	19.3-22.7	109	24.79	3.16	18-34.7	24.7	22.5-27.2	0.000*
InfraM2 (R)	91	21.55	4.18	12.2-30.9	22.2	17.9-24.6	109	23.88	4.06	16.4-32	23.5	20.6-26.6	0.001*
InfraM2 (L)	91	21.39	4.11	11.4-31	21.7	18.2-24	109	24.43	4.26	16-34.8	23.4	21.6-27.9	0.000*

SD -Standard Deviation; IQ-interquartile range; Mann-Whitnet U test; ^*^Significant (P < 0.05).

Descriptive statistics of FSTT, classified by sex and BMI, are presented in Tables 9,10. In males with BMI less and more than 25, the thickness of the tissues differed significantly at all landmarks (*P* < 0.05), except for mp, sd, and id. The thickness of the tissues in these facial landmarks increased as the BMI values increased. The same pattern was observed in females at all reference landmarks except for mp, sd, id and iM2 (r,l) (*P* < 0.05).

**Table 10:** Descriptive statistics of the facial soft tissue thicknesses of Azerbaijan adult population, classified by BMI in females (all the measurements in millimeter).

Landmarks	Females BMI < 25						Females BMI ≥ 25						P-value
	N	Mean	SD	Min-Max	Median	IQ range	N	Mean	SD	Min-Max	Median	IQ range	
Supraglabella	46	5.18	1.06	3.5-8.9	5.1	4.5-5.5	47	5.82	1.19	3-8.1	5.8	4.9-6.8	0.005*
Glabella	46	5.59	1.25	3.9-9.1	5.3	4.9-6.2	47	6.29	1.25	4-8.9	6.2	5.2-7.4	0.004*
Nasion	46	6.03	1.27	3.7-9.5	6	5.2-6.7	47	6.90	1.37	4.9-10.3	6.8	5.7-7.7	0.003*
Rhinion	46	2.50	0.56	1.5-4.2	4.6	2.1-2.9	47	3.08	0.85	1.8-5.9	3	2.4-3.5	0.000*
Mid-philtrum	46	13.13	2.17	8.4-17.3	13.05	11.5-14.7	47	12.47	2.23	8.2-16.9	12.4	10.7-14.4	0.113
Supradentale	46	9.57	1.57	7-14.3	9.45	8.6-10.7	47	10.06	2.01	6-16.6	9.7	8.7-11.2	0.255
Infradentale	46	11.12	2.50	7.3-17.7	10.4	9.4-12.7	47	11.65	2.68	7.6-17.9	11.2	9.4-14.4	0.348
Mentolabial sulcus	46	11.49	1.64	7.1-16.6	11.5	10.8-12.2	47	12.77	2.01	9.1-18.4	12.6	11.7-13.7	0.001*
Pogonion	46	10.14	1.61	6.8-13.4	10.25	9.1-11.2	47	11.73	2.41	7.2-20.1	11.9	10.1-12.9	0.000*
Gnation	46	6.06	1.48	3.8-11	6	5.1-6.8	47	7.90	2.49	3.2-14.6	7.4	6.2-9.4	0.000*
Frontal eminence (R)	46	3.56	0.68	2.3-5.1	3.6	3.1-4	47	4.60	1.67	2.4-10.5	4.3	11.7-13.7	0.000*
Frontal eminence (L)	46	3.45	0.68	2.3-5.1	3.45	2.9-3.8	47	4.51	1.76	2.3-10.9	4	10.1-12.9	0.000*
Supraorbital (R)	46	5.92	1.09	3.2-9.1	5.85	5.2-6.7	47	7.51	1.61	4.7-11.7	7.5	6.2-9.4	0.000*
Supraorbital (L)	46	5.74	1.10	3.1-8.8	5.65	5-6.4	47	7.32	1.59	4.4-12.1	7	3.5-5.3	0.000*
Infraorbital (R)	46	5.02	1.54	2-9.3	5	4.1-5.8	47	6.80	2.23	3.6-13	6	3.5-5.2	0.000*
Infraorbital (L)	46	4.89	1.42	2-8.7	4.9	3.9-5.7	47	6.77	2.77	3.8-17.9	5.9	6.5-8.6	0.000*
Inferior malar (R)	46	12.18	2.31	7.4-16.3	12.1	10.3-14.1	47	14.10	2.92	7.7-19.2	13.8	6.3-8.3	0.001*
Inferior malar (L)	46	11.90	2.41	7.2-16.2	12.2	9.5-13.7	47	14.14	3.27	7.2-21.9	13.7	5-8.3	0.001*
Zygion (R)	46	9.65	1.72	5.6-13.8	9.8	8.8-10.9	47	12.91	2.70	9-20.2	12.2	4.9-8	0.000*
Zygion (L)	46	9.64	1.76	6.3-12.9	9.6	8.5-11	47	13.16	2.92	8.6-20.9	12.8	12.1-16.4	0.000*
Condylion (R)	46	11.19	1.50	7.7-15	11	10.2-12.3	47	13.74	2.30	9-19.1	13.3	11.6-16.5	0.000*
Condylion (L)	46	11.14	1.54	7.6-14.9	11.2	9.9-12	47	13.70	2.19	10.1-18.9	13.4	10.9-14.9	0.000*
Gonion (R)	46	12.38	2.64	7.5-19.4	12.35	10.5-14.3	47	16.44	3.71	8.9-24.4	16	10.7-15	0.000*
Gonion (L)	46	12.04	2.53	7.9-19	11.9	10-13.2	47	16.28	3.71	8.9-23.7	16.5	12.3-15.1	0.000*
SupraM2 (R)	46	25.25	3.86	15.8-32.8	25.6	23.4-27.7	47	29.24	4.88	17.9-40	29.4	12.2-15.2	0.000*
SupraM2 (L)	46	24.85	3.98	14.9-32.5	25.15	22.6-26.8	47	29.86	4.83	20.7-41.4	29.4	14-18.7	0.000*
Occlusal line (R)	46	21.14	2.59	16.1-26.2	21.55	18.9-22.8	47	24.26	3.08	18-32.5	23.8	13.7-19	0.000*
Occlusal line (L)	46	20.87	2.55	15-25.9	21.5	18.8-22.6	47	24.15	2.88	18-30.1	24.2	26.1-32.2	0.000*
InfraM2 (R)	46	21.32	3.94	12.2-28.4	22.05	18.1-24.3	47	22.43	3.60	16.4-31	22.3	26.1-33.5	0.358
InfraM2 (L)	46	21.08	4.13	11.4-29.8	21.6	18.2-23.39	47	22.86	3.80	16-34.8	22.4	22.6-26.3	0.092

SD -Standard Deviation; IQ-interquartile range; Mann-Whitnet U test; ^*^Significant (P < 0.05).

**Table 9:** Descriptive statistics of the facial soft tissue thicknesses of Azerbaijan adult population, classified by BMI in males (all the measurements in millimeter).

	Males BMI < 25						Males BMI ≥ 25						P-value
Landmarks	N	Mean	SD	Min-Max	Median	IQ range	N	Mean	SD	Min-Max	Median	IQ range	
Supraglabella	45	5.15	0.90	3.2-7.9	5	4.7-5.5	62	6.13	1.11	4.2-9.3	5.95	5.3-7	0.000*
Glabella	45	5.77	1.01	4.1-7.9	5.7	4.9-6.5	62	6.47	0.97	4.7-8.4	6.4	5.9-7.1	0.001*
Nasion	45	7.38	1.35	4.3-11.2	7.3	6.4-8.3	62	8.34	1.43	6.1-12.3	8.2	7.2-9.2	0.001*
Rhinion	45	2.93	0.64	2-4.5	5.3	2.4-3.4	62	3.58	0.90	1.8-6	3.5	2.9-4.1	0.000*
Mid-philtrum	45	15.63	2.70	5.3-20.4	15.8	14.1-17.4	62	15.75	2.54	9-20.7	15.6	14.5-17.8	0.855
Supradentale	45	12.28	1.99	8.9-17	12	10.7-13.8	62	12.51	2.00	9.2-16.8	12.4	10.9-14.2	0.620
Infradentale	45	12.90	2.67	7.6-18.6	12.9	11.1-14.6	62	12.84	2.48	8.2-19.7	12.15	11.3-14.1	0.776
Mentolabial sulcus	45	12.85	2.21	8.6-17.4	12.9	11.3-14.3	62	14.64	2.19	10.4-20	14.7	13.3-15.8	0.000*
Pogonion	45	11.03	1.94	7.8-15.8	11.1	9.3-12.6	62	13.21	1.94	8.7-17.4	12.95	11.9-14.8	0.000*
Gnation	45	7.52	1.93	3.8-12.4	7.4	6.4-8.8	62	10.42	2.28	5.8-18.5	10	9.2-11.9	0.000*
Frontal eminence (R)	45	4.34	1.94	2.6-15.5	4	3.6-4.6	62	5.20	1.33	3.2-10.1	4.85	4.2-6.1	0.000*
Frontal eminence (L)	45	4.35	1.91	2.6-14.8	4.1	3.5-4.6	62	5.04	1.34	2.9-9.8	4.8	4-5.9	0.000*
Supraorbital (R)	45	6.38	1.06	4.1-10	6.3	6-6.9	62	7.58	1.21	4.6-10.2	7.55	6.7-8.5	0.000*
Supraorbital (L)	45	6.24	1.07	4.1-9.5	6.2	5.7-6.9	62	7.49	1.20	4.6-10.2	7.4	6.7-8.2	0.000*
Infraorbital (R)	45	4.58	1.38	3.2-10.4	4.2	3.7-5	62	5.82	1.48	3-9.9	5.75	4.7-6.8	0.000*
Infraorbital (L)	45	4.53	1.43	2.6-10.7	4.2	3.6-4.9	62	5.72	1.47	3.1-9.7	5.65	4.7-6.7	0.000*
Inferior malar (R)	45	12.59	2.90	5.3-18.7	12.7	10.6-14.6	62	15.50	2.97	9.2-20.5	15.9	13.4-18	0.000*
Inferior malar (L)	45	12.72	2.90	5.3-18.9	13.2	10.9-14.6	62	15.16	2.96	8.1-21.4	15.15	13.1-17.7	0.000*
Zygion (R)	45	9.67	2.75	4.7-15.9	9.1	7.9-11.2	62	13.48	2.62	7.9-21.2	13.05	12-15.1	0.000*
Zygion (L)	45	9.81	2.67	4.9-15.9	9.5	8.3-11.1	62	13.84	2.92	7.7-21.9	13.45	11.9-15.6	0.000*
Condylion (R)	45	11.72	1.82	7.6-15.7	12.2	10.6-12.7	62	14.75	1.87	10.5-20.7	14.75	13.4-16.2	0.000*
Condylion (L)	45	11.87	1.93	7.5-16.1	11.9	10.9-12.9	62	14.50	1.85	10.2-18.4	14.6	13.1-16	0.000*
Gonion (R)	45	12.54	3.92	4.5-22.1	12.4	9.9-14.5	62	18.31	3.80	10.6-28.5	18.4	15.4-21.3	0.000*
Gonion (L)	45	12.19	3.54	4.7-20.8	11.9	9.5-14	62	18.62	4.10	8.4-30.6	18.4	15.7-21.3	0,000*
SupraM2 (R)	45	26.27	4.75	17.4-41.4	26	23.7-28.3	62	30.37	4.35	17.3-37.5	30.85	28.5-33.7	0.000*
SupraM2 (L)	45	26.80	4.66	18.5-41.1	26.2	24.3-28.2	62	30.42	4.31	18.7-38.4	31.15	28.6-33.3	0.000*
Occlusal line (R)	45	2156	3.05	15.6-28.8	21.8	19.5-23.9	62	25.07	3.46	19.1-34.2	25.25	22.6-26.9	0.000*
Occlusal line (L)	45	21.71	2.90	15.3-28.7	21.9	19.6-23.7	62	25.27	3.31	19.1-34.7	24.75	22.9-27.7	0.000*
InfraM2 (R)	45	21.79	4.44	12.5-30.9	22.2	17.9-25.3	62	24.97	4.07	17.1-32	25.85	22-27.7	0.000*
InfraM2 (L)	45	21.71	4.10	12.4-31	22	19.5-24.2	62	25.62	4.23	16.9-33.7	25.8	22.6-28.4	0.000*

SD -Standard Deviation; IQ-interquartile range; Mann-Whitnet U test; ^*^Significant (P < 0.05).

Descriptive statistics of FSTT, classified by age and BMI, are presented in Tables 11–13. When the FSTT data of the first age group were analysed, statistically significant differences were observed between the categories of underweight, normal (BMI less than 25), and overweight, obesity (BMI more than 25) for all landmarks. In individuals with a BMI greater than 25, the facial soft tissues were thicker in all landmarks, except for sg, g, mf, id, cld (l), and iM2 (r,l), which was statistically confirmed. A difference of more than 4 mm in the thickness of the facial soft tissues was determined in the area of gnathion (+ 4.25 мм), zygion,l (+ 4.29 мм), condylion,r (+ 5.41 мм), condylion,l (+ 5.41 мм), gonion,r (+ 4.56 мм), gonion,l (+ 4.70 мм).

**Table 11:** Descriptive statistics of the facial soft tissue thicknesses of Azerbaijan adult population, classified by BMI of I age group (18-25 years) (all the measurements in millimeter).

Landmarks	BMI < 25 (n = 28)					BMI ≥ 25 (n = 5)					P-value
	Mean	SD	Min-Max	Median	IQ range	Mean	SD	Min-Max	Median	IQ range	
Supraglabella	5.07	0.78	3.5-7.3	4.95	4.55-5.5	5.96	1.24	4.5-7.3	6.1	4.9-7	0.138
Glabella	5.70	1.13	4-8.4	5.65	4.8-6.55	6.64	0.91	5.7-8	6.2	6.2-7.1	0.087
Nasion	6.83	1.35	4.2-11.2	6.7	6.15-7.5	9.54	1.29	8.1-11.6	9.4	9-9.6	0.002^*^
Rhinion	2.57	0.57	1.5-4.2	2.4	2.25-2.9	3.36	0.59	2.6-4	3.2	3.1-3.9	0.012*
Mid-philtrum	15.53	2.17	11.8-20.4	15.55	13.95-17.05	16.22	2.12	13.2-19.1	16	15.9-16.9	0.564
Supradentale	11.69	1.76	7.9-14.4	11.25	10.45-13.3	13.72	0.96	12.6-15	13.3	13.3-14.4	0.022*
Infradentale	11.88	2.70	7.8-18.1	12	9.5-14.05	11.64	1.80	9.1-13.9	11.4	11.1-12.7	0.841
Mentolabial sulcus	12.19	2.22	7.1-17	12	10.85-13.6	15.20	2.64	13.3-19.8	14.6	13.6-14.7	0.015*
Pogonion	10.59	1.81	7-13.7	10.6	9.4-11.6	13.72	2.10	11.9-17.1	13.4	12.1-14.1	0.005*
Gnation	7.01	1.95	3.9-11.6	6.7	5.5-8.55	11.26	2.28	9.1-14.6	10.1	9.9-12.6	0.001*
Frontal eminence (R)	3.81	0.63	2.3-5	3.8	3.6-4.15	5.54	1.10	4.6-7.3	5.6	4.6-5.6	0.002*
Frontal eminence (L)	3.82	0.78	2.3-6.2	3.65	3.5-4.15	5.36	1.09	4.2-7.1	5	4.9-5.6	0.003*
Supraorbital (R)	6.08	0.83	4.4-7.6	6.1	5.4-6.75	7.96	1.36	6.5-9.5	7.4	7.1-9.3	0.005*
Supraorbital (L)	5.84	0.94	4.1-7.3	6	5.1-6.55	7.84	1.77	6.4-10.1	6.8	6.5-9.4	0.013*
Infraorbital (R)	4.28	0.73	3.2-5.8	4.15	3.7-4.85	5.58	0.56	4.6-6	5.8	5.7-5.8	0.003*
Infraorbital (L)	4.19	0.76	2.6-5.7	4.2	3.6-4.7	5.28	0.51	4.5-5.8	5.4	5.1-5.6	0.005*
Inferior malar (R)	11.43	2.28	5.3-15	11.65	9.75-13.4	15.10	3.36	10-19.3	15.4	14.6-16.2	0.014*
Inferior malar (L)	11.55	2.37	5.3-15	11.9	9.6-13.55	14.66	3.94	8.1-18.3	15.2	14.7-17	0.037*
Zygion (R)	9.02	1.78	4.712.2	9.1	7.85-10.55	12.64	3.37	8.1-16.4	13.8	10.3-14.6	0.035*
Zygion (L)	8.95	1.65	5-12.6	8.9	7.95-10.1	13.24	3.36	7.7-16.4	14.6	12.7-14.8	0.018*
Condylion (R)	11.35	1.54	8-15	11.6	10.1-12.3	12.82	1.53	10.5-14.5	12.9	12.4-13.8	0.039*
Condylion (L)	11.24	1.62	7.6-14.4	11.15	10.2-12.4	12.70	1.53	10.2-14.3	12.9	12.7-13.4	0.063
Gonion (R)	12.09	2.85	7.5-18.3	12.15	9.7-13.75	17.50	2.45	14.8-20.7	17.7	15.4-18.9	0.002*
Gonion (L)	11.77	3.00	7-18	11.75	9.4-13.4	17.18	2.16	14.7-20.5	16.7	16.2-17.8	0.004*
SupraM2 (R)	25.46	3.91	15.8-35.7	25.45	23.95-27.35	30.02	3.51	25.1-34.8	30.3	28.9-31	0.021*
SupraM2 (L)	25.78	4.11	14.9-35.9	26.05	24.7-27,85	30.48	3.72	24.6-34.9	30.8	30.5-31.6	0.033*
Occlusal line (R)	21.49	2.66	16.1-26.1	21.75	19.75-23.8	25.32	3.15	21.2-28	26.9	22.7-27.8	0.031*
Occlusal line (L)	21.41	2.80	15-28.7	21.85	19.95-22.7	25.40	2.38	22.1-27.7	26.2	23.8-27.2	0.012*
InfraM2 (R)	22.62	4.04	12.2-30.9	22.7	21.55-25.3	24.90	4.72	20.4-32	22.8	22-27.3	0.530
InfraM2 (L)	22.29	4.17	11.4-31	23	19.85-24.75	26.04	4.22	21.8-32.2	24.5	23.3-28.4	0.114

SD -Standard Deviation; IQ-interquartile range; Mann-Whitnet U test; ^*^Significant (P < 0.05).

**Table 12:** Descriptive statistics of the facial soft tissue thicknesses of Azerbaijan adult population, classified by BMI of II age group (26-45years) (all the measurements in millimeter).

Landmarks	BMI < 25 (n = 49)					BMI ≥ 25 (n = 48)					P-value
	Mean	SD	Min-Max	Median	IQ range	Mean	SD	Min-Max	Median	IQ range	
Supraglabella	5.29	1.05	3.6-8.9	5.1	4.7-5.5	5.83	1.20	3-9.3	5.8	5-6.8	0.007^*^
Glabella	5.78	1.17	3.9-9.1	5.4	4.9-6.5	6.21	1.14	4-8.3	6.05	5.15-7.4	0.050*
Nasion	6.58	1.51	3.7-10	6.4	5.6-7.6	7.73	1.70	5-12.3	7.5	6.45-9	0.001*
Rhinion	2.62	0.55	1.8-4.3	2.6	2.2-2.9	3.22	0.90	1.8-5.9	3.1	2.6-3.7	0.000*
Mid-philtrum	13.87	2.75	5.3-19.3	13.8	12.4-15.8	15.14	2.83	9-20.7	15.2	13.5-16.7	0.032*
Supradentale	10.38	2.04	7.1-16.8	9.9	8.9-11.1	11.69	2.17	8.2-16.8	11.4	9.85-13.1	0.002*
Infradentale	11.75	2.59	7.3-17.7	11.3	10-13.2	12.65	2.78	8.5-19.7	11.75	10.75-14.65	0.103
Mentolabial sulcus	12.21	1.86	7.5-16.6	12.1	11.3-13	13.60	2.60	9.1-19.4	13.1	11.8-15.5	0.009*
Pogonion	10.75	1.84	7.4-15.8	10.9	9.5-11.7	12.35	2.65	7.2-20.1	12.25	10.5-14.15	0.002*
Gnation	6.74	1.88	3.8-12.4	6.6	5.5-7.5	9.15	2.80	3.2-15.1	9.3	7.05-11.4	0.000*
Frontal eminence (R)	4.08	1.89	2.3-15.5	3.8	3.3-4.4	4.88	1.40	3.1-10.1	4.65	3.8-5.75	0.000*
Frontal eminence (L)	4.00	1.85	2.4-14.8	3.7	3.2-4.2	4.68	1.36	2.5-9.8	4.4	3.75-5.4	0.000*
Supraorbital (R)	6.23	1.14	3.2-10	6.1	5.4-6.8	7.24	1.26	4.6-10.2	7.3	6.45-8.15	0.000*
Supraorbital (L)	6.12	1.13	3.1-9.5	6	5.5-6.6	7.08	1.21	4.4-10.2	7.1	6.4-7.85	0.000*
Infraorbital (R)	5.11	1.60	2.3-10.4	4.9	4.1-5.8	5.89	1.69	3.3-12,1	5.75	4.7-6.9	0.011*
Infraorbital (L)	5.03	1.53	2.3-10.7	4.8	4.1-5.7	5.78	1.59	3.4-11.7	5.5	4.7-6.7	0.011*
Inferior malar (R)	12.87	2.75	7.4-18.7	13	11-15.1	15.04	2.93	8.6-20.5	15.3	13.2-17.2	0.001*
Inferior malar (L)	12.71	2.86	7.2-18.9	13	11-14.7	14.81	2.87	7.2-19.9	14.9	13.1-17.25	0.001*
Zygion (R)	10.17	2.55	5.1-15.9	9.8	8.7-11.5	13.38	2.57	7.9-21.2	13.35	11.85-14.75	0.000*
Zygion (L)	10.32	2.46	4.9-15.9	9.6	8.8-11.9	13.69	2.91	8.1-21.9	13.4	11.8-15.45	0.000*
Condylion (R)	11.52	1.81	7.6-15.7	11.4	10.3-12.6	14.04	1.93	10-20.7	14.05	13-15.3	0.000*
Condylion (L)	11.62	1.95	7.5-16.1	11.5	10.3-12.5	13.91	1.71	10.7-17.8	14	12.65-15.15	0.000*
Gonion (R)	12.50	3.54	4.5-22.1	12.6	10.5-14.3	17.60	3.88	8.9-28.5	17.45	15.15-21.15	0.000*
Gonion (L)	12.23	3.17	4.7-20.8	11.9	10.2-13.8	18.05	4.57	8.4-30.6	18	14.85-21.3	0.000*
SupraM2 (R)	26.05	4.63	17-41.4	25.8	24-27.7	30.38	4.05	17.3-37.5	30.65	28.35-33.55	0.000*
SupraM2 (L)	26.00	4.76	16.1-41.1	25.2	23.7-28.1	30.39	3.80	18.7-37.7	30.15	28.4-32.7	0.000*
Occlusal line (R)	21.50	2.97	15.6-28.8	21.4	19.3-23.5	24.91	3.35	18-34.2	24.95	22.75-26.55	0.000*
Occlusal line (L)	21.51	2.75	15.3-26.7	21.8	19.3-23.6	25.07	3.26	18-34.7	24.8	22.9-27.5	0.000*
InfraM2 (R)	21.22	4.20	12.5-28.6	21.2	18.1-24.3	25.09	3.73	16.4-31.6	25.85	23-27.2	0.000*
InfraM2 (L)	21.14	4.21	12-29.8	21.1	18.5-23.9	25.40	3.99	16.8-33.7	25.65	22.45-28.3	0.000*

SD-Standard Deviation; IQ-interquartile range; Mann-Whitnet U test; ^*^Significant (P < 0.05).

**Table 13:** Descriptive statistics of the facial soft tissue thicknesses of Azerbaijan adult population, classified by BMI of III age group (46 and above) (all the measurements in millimeter).

Landmarks	BMI < 25 (n = 14)					BMI ≥ 25 (n = 56)					P-value
	Mean	SD	Min-Max	Median	IQ range	Mean	SD	Min-Max	Median	IQ range	
Supraglabella	4.94	1.07	3.2-7.2	4.95	4.4-5.4	6.14	1.11	4.1-8.1	6.25	5.3-6.9	0.002*
Glabella	5.29	1.00	3.9-7.7	5.2	4.8-6	6.52	1.07	4.2-8.9	6.5	5.8-7.05	0.000*
Nasion	6.84	1.62	4.3-9.1	6.7	6-8.6	7.55	1.38	4.9-11.1	7.5	6.5-8.4	0.179
Rhinion	3.31	0.77	1.9-4.5	3.4	2.7-3.9	3.49	0.93	1.8-6	3.4	2.9-3.9	0.752
Mid-philtrum	13.77	3.17	8.4-19.7	13.15	11.4-15.7	13.48	2.78	8.2-19.8	13.35	11.6-15.25	0.849
Supradentale	11.24	3.24	7-17	10.7	8-13.8	11.04	2.44	6-16.6	10.85	9.35-12.25	0.982
Infradentale	13.10	3.11	8.4-18.6	12.8	10.9-15.7	12.11	2.55	7.6-18	12	10.25-14.1	0.301
Mentolabial sulcus	11.96	2.45	8.2-17.4	11.3	10.3-13.1	13.91	1.98	10-20	13.65	12.5-15.1	0.005*
Pogonion	9.96	1.78	6.8-12.6	10	8.5-11.5	12.66	1.90	8.7-17.4	12.55	11.7-13.65	0.000*
Gnation	6.44	1.64	3.8-10.2	6.4	5.6-7.2	9.33	2.57	5.1-18.5	9.3	7.45-10.7	0.000*
Frontal eminence (R)	3.76	1.12	2.6-6.4	3.25	2.9-4.5	4.94	1.64	2.4-10.5	4.55	3.8-5.6	0.004*
Frontal eminence (L)	3.66	1.13	2.6-6.3	3.15	2.9-4.4	4.87	1.73	2.3-10.9	4.4	3.8-5.6	0.005*
Supraorbital (R)	6.02	1.42	4.1-9.1	6.15	4.7-6.9	7.78	1.47	5.1-11.7	7.85	6.7-8.8	0.000*
Supraorbital (L)	5.80	1.33	4.2-8.8	5.8	4.7-6.5	7.66	1.44	4.8-12.1	7.55	6.75-8.55	0.000*
Infraorbital (R)	4.75	1.86	2-9.3	4.5	3.3-5.6	6.61	2.07	3-13	6.05	5.2-8.2	0.002*
Infraorbital (L)	4.66	1.82	2-8.4	4.3	3.3-5.7	6.59	2.60	3.1-17.9	5.95	4.9-7.55	0.006*
Inferior malar (R)	12.60	2.37	7.4-17.3	12.45	11.4-14.4	14.75	3.12	7.7-20	14.7	12.05-17.4	0.022*
Inferior malar (L)	12.41	2.45	7.2-17.2	12.8	11-13.6	14.64	3.31	8.9-21.9	14.35	12-17.4	0.036*
Zygion (R)	9.15	1.78	6.6-12.5	9.2	7.6-10.3	13.16	2.71	9-20.2	12.7	11-15	0.000*
Zygion (L)	9.17	1.98	6.3-12.5	9.4	7.3-11	13.46	2.94	8.6-20.9	12.85	11.25-15	0.000*
Condylion (R)	11.44	1.59	8.6-14.2	11.9	10-12.5	14.69	2.25	9-19.1	14.9	12.7-16.55	0.000*
Condylion (L)	11.59	1.44	9.5-14.4	11.7	10.2-10.5	14.49	2.26	10.1-18.9	14.7	12.65-16.2	0.000*
Gonion (R)	13.03	3.51	8.2-19.4	13.2	9.9-15.2	17.42	3.99	9.4-24.9	17.6	14.95-20.25	0.001*
Gonion (L)	12.40	2.91	9-19	12.9	9.3-14	17.27	3.78	10-24.7	17.9	14.15-20.2	0.000*
SupraM2 (R)	25.29	4.28	16.9-30.6	26	23.3-29.1	29.45	5.12	17.9-40	30.15	26.05-32.6	0.004*
SupraM2 (L)	25.27	3.97	18.2-32.5	25.75	23-27.3	29.97	5.18	20-41.4	30.9	25.9-33.65	0.003*
Occlusal line (R)	20.54	2.67	16.3-24.1	21.65	17.4-22.5	24.50	3.34	18.4-32.5	24	22.25-26.85	0.000*
Occlusal line (L)	20.27	2.57	16.3-24	20.7	17.4-22.3	24.49	3.15	18.8-32.8	24.35	22.15-26.8	0.000*
InfraM2 (R)	20.58	4.26	12.7-26.4	21.8	16.6-24.3	22.75	4.02	17.1-31.9	22.25	19.8-25.2	0.201
InfraM2 (L)	20.51	3.55	15.3-26.3	21.6	16.9-22	23.45	4.32	16-34.8	22.65	20.15-26.15	0.043*

SD -Standard Deviation; IQ-interquartile range; Mann-Whitnet U test; ^*^Significant (P < 0.05).

In the second age group, the FSTT in the area of 14 anthropometric landmarks was greater in individuals with overweight and obesity. At the same time, in the area of id landmark did not reveal statistically significant differences between the groups with BMI less and more than 25. A difference of more than 4 mm in FSTT was determined in the area of gonion,r (+ 5.10 мм), gonion,l (+ 5.82 мм), supraM2,r (+ 4.33 мм), supraM2,l (+ 4.39 мм), infra M2,l (+ 4.27 мм).

In the third age group, in individuals with overweight and obesity, the thickness of the facial soft tissues was greater in the area of 14 anthropometric landmarks, which was statistically confirmed. A difference of more than 4 mm in thickness of facial soft tissues was determined in the area of zygion,r (+ 4.01мм), zygion,l (+ 4.29 мм), gonion,r (+ 4.39 мм), gonion,l (+ 4.87 мм), supraM2,r (+ 4.16 мм), supraM2,l (+ 4.70 мм), occlusial line,l (+ 4.22 мм).

## Discussion and conclusion

Studies on the thickness of facial soft tissues have been conducted since the end of the 19th century. Measurement of FSTT is performed by forensic scientists, maxillofacial and plastic surgeons, orthodontists, and anthropologists.

Numerous studies have confirmed the presence of FSTT variations among populations, indicating the importance of using specific values for each population [27,32,40,55–57]. Thieman, by comparing databases, came to the conclusion that population specificity influences on FSTT [31]. However, scientific papers have been published that contradict or do not confirm this hypothesis [31,38,58]. This could be explained by the fact that the data of the average FSTT values obtained using different measurement protocols were compared [59].

The development of non-invasive diagnostic methods, such as cephalometric radiography [21,28,30,42,43,45,48,49,53], ultra-sound examination [7,20,26,28,33,35,36,54], magnetic resonance imaging [23,28,39,41,49], and computed tomography [13–15,27,29,31,34,37,38,40,55,57,60], has made it possible to conduct studies on the thickness of facial soft tissues *in vivo*. However, invasive studies of FSTT *ex vivo* using the puncture method are still ongoing [3,19,24,25,32,44,54,55].

The method of collecting soft tissue thickness data from CT images is considered to be one of the most accurate; however, it has a high output radiation power, which is harmful to biological tissues. CT of the paranasal sinuses is often performed for diagnostic purposes, which contributes to the collection of head CT-scans. Concurrently, the CT data stored in the archives of radiological departments of clinics can be utilised to study and create a large FSTT database.

Thus, Connie L. Parks conducted a study of the thickness of the facial soft tissues of Americans on CT images of 388 patients aged from 18 to 62 years, taking into account their BMI. Furthermore, Panenkova carried out a study of FSTT on CT images of 160 adult patients of the Slovak population aged 18–60 years, without consideration of the BMI. In addition, Pierre Guyomarc’h conducted a study on the thickness of facial soft tissues in representatives of the French population also on CT images. The study included 500 people aged 18–96 years, taking into account their BMI. Moreover, Anna Drga´ cˇova´ performed a study of FSTT on 102 CT images of representatives of the Czech population aged 21–83 years, without consideration of the BMI. Thiemann N analysed data on the modern adult population of Germany, on the material of 320 people according to CT, taking into account sex, age and BMI. Based on the CT images of 320 individuals, Bulut O. conducted a study of the FSTT of the Turkish population, taking into account sex and age; patients only with normal weight were included in the study. A study of the FSTT of representatives of the Chinese population was performed by Ya Dong using CT images of 200 patients aged 18–32 years, taking into account sex and BMI. Furthermore, Cavanagh D. employed CT scanning to determine the average FSTT values in 154 South African black females. FSTT of the 100 representatives of the Korean population aged 20–36 years was studied on CT images, by H. Hwang. Furthermore, Moritsugui D.S., investigated the soft tissues thickness of 101 representatives of the Brazilian population of three age groups, from 18 and above, taking into account age and gender. The study of FSTT on CT scans of 75 the Bulgarian adults aged 20–74 years was carried out by Toneva D, representatives were divided into groups according to sex, age and BMI. Based on these studies, a data bank of FSTT was created for these populations [13,31,34,37,38,40,46,55,60].

A literature review revealed the absence of a database of FSTT in the Azerbaijan population. In this study, FSTT was investigated on CT scans of 300 individuals, taking into account sex, age and BMI, on the basis of which a database was created and a number of conclusions were made.

This study demonstrated a statistically significant difference in FSTT between the sexes, and sexual dysmorphism in the Azerbaijan population was confirmed. The greatest difference was observed in the upper lip and chin. The thickness of the facial soft tissues in males was greater than that in females. Similar results were obtained in numerous studies [3,13,19,31].

A study of the relationship between FSTT and age revealed thickening of the facial soft tissues with age at all the anthropometric landmarks. Gradual thickening of the FSTT was observed in the area of studied landmarks. The exception was the upper lip, the thickness of the soft tissues of which decreases with age.

Comparison of the thickness of facial soft tissues in males and females of different age groups showed that at the age of 18–25 years and 46 years and above, differences were observed only in the middle and lower part of the face, whereas at the age of 26–45 years, differences were found in the upper, middle, and lower part of the face. However, Guyomarc’h P. in his work revealed an insignificant difference between age groups [38].

During the analysis of the obtained information, a correlation was revealed between the thickness of the facial soft tissues and the BMI for all reference landmarks. The thickness of the tissues in the area of the facial anthropometric landmarks increased as the BMI increased, with the exception of the projection of the upper and lower lips [13,23,26,31,38].

Our study also showed that with age, the effect of BMI on the thickness of facial soft tissues persists, which was observed in all three age groups.

The creation of data banks that use facial tissue thickness data classified according to race, sex, age, BMI will provide forensic scientists, plastic and maxillofacial surgeons, and anthropologists with more accurate information.

This article presents the first database of the thickness of facial soft tissues of Transcaucasia populations. The data from this study can be used to update pooled means and create a new version of the Global T-Table.

### Limits of the study and further recommendations

CT examination of the FSTT in our study were done with patients in the supine position. The gravity is the limitation of this method of measuring of FSTT. In differ from this scientific work, the future studies of azerbaijan population could benefit from examination of the relationship between age, sex, BMI and the measurement of FSTT with upright position of the head.

Measurements of FSTT in gonion landmark, taken in the coronal plane may occasionally result in a non-perpendicular orientation to the bone surface, due to the bone’s angular orientation.

## Supporting information

S1 Table All raw data required to replicate the results of this study. (XLSX)
